# Compartment Syndrome Following Snake Envenomation in the United States: A Scoping Review of the Clinical Literature

**DOI:** 10.5811/westjem.18401

**Published:** 2024-06-14

**Authors:** John Newman, Colin Therriault, Mia S. White, Daniel Nogee, Joseph E. Carpenter

**Affiliations:** * Emory University School of Medicine, Department of Emergency Medicine, Atlanta, Georgia; † Vanderbilt University Medical Center, Department of Pathology, Microbiology and Immunology, Nashville, Tennessee; ‡ University of Illinois College of Medicine, Department of Emergency Medicine, Peoria, Illinois; § Woodruff Health Sciences Center Library, Emory University, Atlanta, Georgia; ∥ Georgia Poison Center, Atlanta, Georgia

## Abstract

**Introduction:**

Local tissue destruction following envenomation from North American snakes, particularly those within the Crotalinae subfamily, has the potential to progress to compartment syndrome. The pathophysiology of venom-induced compartment syndrome (VICS) is a debated topic and is distinct from trauma/reperfusion-induced compartment syndrome. Heterogeneity exists in the treatment practices of VICS, particularly regarding the decision to progress to fasciotomy. Associations with functional outcomes and evolution in clinical practice since the introduction of Crotalidae polyvalent immune Fab (FabAV) have not been well defined. Our goal was to identify the potential gaps in the literature regarding this phenomenon, as well as illuminate salient themes in the clinical characteristics and treatment practices of VICS.

**Methods:**

We conducted this systematic scoping-style review using the Preferred Reporting Items for Systematic Reviews and Meta-Analyses (PRISMA) guidelines. Records were included if they contained data surrounding the envenomation and hospital course of one or more patients who were envenomated by a snake species native to North America and were diagnosed with compartment syndrome from 1980–2020.

**Results:**

We included 19 papers: 10 single- or two-patient case reports encompassing 12 patients, and nine chart reviews providing summary statistics of the included patients. In case reports, the median compartment pressure when reported was 60 millimeters of mercury (interquartile range 55–68), 66% underwent fasciotomy, and functional outcomes varied. Use of antivenom appeared to be more liberal with FabAV than the earlier antivenin Crotalidae polyvalent. Rapid progression of swelling was the most commonly reported symptom. Among the included retrospective chart reviews, important data such as compartment pressures, consistent laboratory values, and snake species was inconsistently reported.

**Conclusions:**

Venom-induced compartment syndrome is relatively rare. Existing papers generally describe good outcomes even in the absence of surgical management. Significant gaps in the literature regarding antivenom dosing practices, serial compartment pressure measurements, and functional outcomes highlight the need for prospective studies and consistent standardized reporting.

Population Health Research Capsule What do we already know about this issue?

*Compartment syndrome is a rare complication of envenomation by certain snake species; clinical data regarding this phenomenon is poorly described.*

What was the research question?

*What are the clinical characteristics, treatment paradigms and functional outcomes of venom-induced compartment syndrome (VICS)?*

What was the major finding of the study?

*For 19 papers, the median compartment pressure was 60 mm Hg (IQR 55–68) and 66% underwent fasciotomy. Functional outcomes varied but were generally good. *

How does this improve population health? (165 characters max)

*This review distills what is known about VICS and highlights important gaps in the literature, including long-term functional outcomes.*

## INTRODUCTION

The venomous snakes of North America capable of causing significant soft tissue damage fall under the family Viperidae and the subfamily Crotalinae (also referred to as crotalids).^1^ Snakes in this category consist of the genera *Crotalus* (rattlesnakes), *Sistrurus* (pygmy rattlesnake and massasauga), and *Agkistrodon* (cottonmouth and copperheads). These genera are often colloquially referred to as pit vipers due to the presence of heat-sensing pits behind their nostrils.^2^ Crotalid venom is a complex mixture of more than 100 macromolecules, glycoproteins, and metals. Phospholipase A2, inflammatory mediator analogues, and metalloproteinases damage endothelium and disrupt normal coagulation cascades, primarily manifesting as local tissue destruction and hematologic toxicity, although neurotoxicity can develop after envenomation from some species.^3^^,^^4^ In severe cases, tissue destruction and swelling due to crotalid envenomation has the potential to progress to compartment syndrome. In contrast, elapid venom found in North American coral snakes results in little to no local tissue destruction.^5^^,^^6^

The nature of venom-induced compartment syndrome (VICS) is a debated topic, as local symptoms common to crotalid envenomation such as pallor, edema, paresthesia, and pain with passive movement can mimic trauma or reperfusion-associated compartment syndrome. However, true compartment syndrome is thought to be rare after envenomation, as the associated symptoms are more likely due to direct myonecrosis rather than elevated compartment pressures and associated tissue ischemia.^3^^,^^5^^–^^7^ As a result, some advocate against using the term compartment syndrome to describe the condition. Consequently, there is heterogeneity in how clinicians approach suspected cases of VICS, including the role of fasciotomy.

This treatment inconsistency also stems from historic misguidance of suspected cases of compartment syndrome following envenomation, which reached its nadir in the 1970s–1980s when fasciotomy was considered the gold standard of treatment. Numerous reviews and animal models suggest that prompt antivenom administration precludes the need for fasciotomy, as antivenom treatment alone has been shown to reduce intracompartmental pressures in animal models.^8^^,^^9^ In a 2011 review, Cumpston concluded that current evidence did not support the use of fasciotomy in Crotalinae envenomation with elevated compartment pressures and might even worsen outcomes.^7^ Of note, the majority of articles included in that review describe patients treated with antivenin Crotalidae polyvalent (ACP), prior to the introduction of Crotalidae polyvalent immune Fab (FabAV), adding significance to an additional review.

At our institution, we were recently consulted in two copperhead envenomations in which local tissue damage progressed to alleged compartment syndrome with elevated compartment pressures; fasciotomy was performed in both cases. This led our team to question how often this clinical scenario occurs and what literature exists to guide management and inform prognosis. Therefore, we performed a review of literature reporting compartment syndrome following North American snake envenomation to gather data regarding symptomatology, laboratory/pressure abnormalities, interventions, and outcomes and to identify gaps in the literature surrounding this phenomenon, particularly concerning functional outcomes.

## METHODS

We conducted a systematic review of published studies reporting compartment syndrome following North American snake envenomation from January 1, 1980–November 18, 2020. Our team included three medical toxicologists, one resident physician, and an information specialist (librarian MSW). We used the Preferred Reporting Items for Systematic Reviews and Meta-Analyses (PRISMA) statement as the guideline for conducting this review.^10^ According to the guidelines of the Emory University Institutional Review Board, this study was not human subjects research and did not require review.

After consultation with other team members, the information specialist developed a search strategy; six databases were searched. We searched terms “compartment syndromes,” “snake bite,” “snake bites,” and “North America.” The systematic searches were performed in Agricola (Ebscohost), Cochrane Central Register of Controlled Trials (CENTRAL via Cochrane Library), Embase (Elsevier), Global Health (CAB Direct), PubMed (NLM), and Web of Science (core collections via Clarivate) databases on November 18, 2020. The complete search terms and strategies are included as supplemental information. We filtered search results for English language and journal articles only; editorials and letters were excluded in each database. In addition, where applicable, we also sought conference abstracts and reviews if we felt that there were sufficient data points within the abstract. During the search process, if there were fewer than five records retrieved, filters such as English language and article type were not applied.

All records (278) were imported into Covidence (Melbourne, Australia), and duplicate citations were removed by Covidence prior to the review. Fifty-four duplicates were removed, and 224 records were set to be screened. Records were eligible for inclusion if they contained demographic and bite-related data regarding one or more patients who were envenomated by a snake species native to North America and were diagnosed with compartment syndrome. One resident and two medical toxicologists reviewed the records, and a third medical toxicologist resolved records in dispute. Results are presented in descriptive and tabular format. No formal statistical analyses were performed.

## RESULTS

After initial screening of the 224 records retrieved, 161 studies were deemed irrelevant, usually due to bites from animals other than snakes, envenomation by a non-native species of snake, or bites that took place outside the United States. Upon review of the complete articles, we excluded an additional 44 due to absence of significant documented outcome measures, leaving 19 studies for study inclusion (Figure). Of the 19 studies included, 10 were single- or two-patient case reports providing patient-level data^11^^–^^20^ (Tables 1–3) and nine were chart review summaries providing summary statistics of the included patients^5^^,^^21^^–^^28^ (Table 4). All species causing VICS in this review fall under the Crotalinae subfamily (crotalids). In total, 88 cases were extracted: 12 from single- or two-patient case reports, and 76 cases from retrospective chart reviews.

**Figure.**
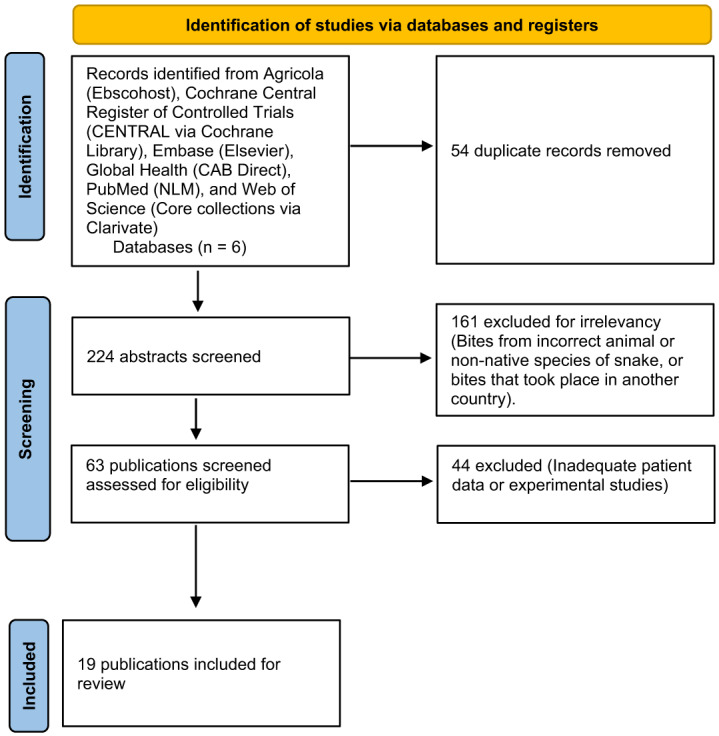
PRISMA flow diagram.

**Table 1.** Demographics for case reports.

Publication	Age (y)	Gender	Snake species	Bite location
Roberts et al, 1985 [13] (patient 1)	14	M	Pygmy rattlesnake	Finger
(Patient 2)	39	M	Cottonmouth	Volar hand
Seiler et al, 1994 [14]	8	M	Not specified	Posterior lower extremity
Padda and Bowen, 1995 [15]	5	M	Copperhead	Ankle
Rosen et al, 2000 [16]	59	M	Western diamondback	Dorsal foot
Gold et al, 2003 [17]	43	M	Western diamondback	Volar hand
Hardy et al, 2006 [18]	35	F	Black-tailed rattlesnake	Anterior lower extremity
Thomas et al, 2011 [19] (Patient 1) (Patient 2)	8	F	Great Basin rattlesnake	Ankle
	2	M	Great Basin rattlesnake	Finger
Mazer-Amirshahi et al, 2014 [20]	1	F	Copperhead	Dorsal foot
Brys et al, 2015 [21]	9	M	Copperhead	Dorsal hand
McBride et al, 2017 [22]	48	M	Eastern diamondback	Anterior lower extremity

*M*, male; *F*, female.

**Table 2.** Symptoms and compartment pressures for case reports.

Publication	Pain (passive)	Edema	Erythema	Rapid swelling	Firm compartment	Other	Pressure (mm Hg)
Roberts et al, 1985 [13] (Patient 1)	Yes	Yes	No	Yes	Yes	Paresthesia, numbness, diminished pulses	60
(Patient 2)	Yes	Yes	No	Yes	Yes	Paresthesia, diminished pulses	60
Seiler et al, 1994 [14]	Yes	Yes	No	Yes	Yes	Paralysis	55
Padda and Bowen 1995 [15]	No	No	No	Yes	Yes	Paresthesia	35
Rosen et al, 2000 [16]	Yes	Yes	No	Yes	Yes	None	46
Gold et al, 2003 [17]	Yes	Yes	Yes	Yes	Yes	Paresthesia	55
Hardy et al, 2006 [18]	Yes	Yes	No	Yes	No	Paresthesia, paralysis	68
Thomas et al, 2011 [19] (Patient 1)	Yes	Yes	Yes	Yes	Yes	None	68
(Patient 2)	No	Yes	No	Yes	Yes	Poikilothermia, weak pulses	60
Mazer-Amirshahi et al, 2014 [20]	Yes	Yes	Yes	Yes	No	None	85
Brys et al, 2015 [21]	Yes	Yes	No	Yes	Yes	None	56
McBride et al, 2017 [22]	No	Yes	No	No	Yes	None	72

*mm Hg*, millimeters of mercury.

**Table 3.** Treatments and outcomes for case reports.

Publication	Antivenom	# of vials	Fasciotomy performed	Length of hospitalization (days)	Outcome	Follow-up time
Roberts et al, 1985 [13] (Patient 1)	Not specified*	7	Yes	4	Complete resolution	4 days
(Patient 2)	Not specified*	10	Yes	5	Complete resolution	5 days
Seiler et al, 1994 [14]	Not specified*	5	Yes	Not specified	Residual motor deficit	1 year
Padda and Bowen 1995 [15]	Not specified*	Not specified	Yes	4	Not specified	N/A
Rosen et al, 2000 [16]	ACP	15	No	2	Pain with walking	1 week
Gold et al, 2003 [17]	ACP	30	No	3	Complete resolution	6 days
Hardy et al, 2006 [18]	FabAV	12	Yes	12	Abscess, motor deficit	2 months, 2 years
Thomas et al, 2011 [19] (Patient 1)	Not specified^#^	32	No	6	Not reported	N/A
(Patient 2)	Not specified^#^	15	Yes	5	Not reported	N/A
Mazer-Amirshahi et al, 2014 [20]	FabAV	26	No	4	Not reported	2 weeks
Brys et al, 2015 [21]	FabAV	16	Yes	Not specified	Not reported	2 weeks
McBride et al, 2017 [22]	FabAV	54	Yes	15	Not reported	N/A

*Assumed to be ACP based on year of publication. ^#^Assumed to be FabAV.

*ACP*, antivenin Crotalidae polyvalent; *FabAV*, Crotalidae polyvalent immune Fab.

**Table 4.** Summary statistics for cumulative data studies.

Study	Methods	Patient/bite characteristics	Signs and symptoms	Treatment(s)	Outcome(s)
Downey et al, 1991 [23]	Single-center, retrospective chart review using orthopedic operation logs and hospital admission records over an 11-year period.	36 patients, 28 (78%) male. Median age 21 years (range 2–71). 5 (14%) foot/ankle bites, 7 (19%) leg bites, 20 (56%) hand bites, 2 (6%) forearm and 2 (6%) upper arm bites. Snake species not recorded. Most common activities before being bitten included alcohol use, playing outside, and handling a pet snake.	Study used modified Wood, Hoback, and Green (McCollough, N and Gennaro, J et al1968) envenomation scale. 5 (14%) grade 1, 27 (75%) grade 2, and 3 (8%) grade 3 bites. Of the 25 (69%) patients diagnosed with compartment syndrome, 7 were in the digit, 9 in the hand/forearm, 1 in the arm, and 8 in the foot/leg. 25 diagnosed with compartment syndrome.	Antivenom used in 22 (61%) of all bites and in 11/15 (73%) of patients under the age of 18, with a total dose ranging from 1–15 vials. Serum sickness occurred in 1 patient receiving antivenom. All 25 patients diagnosed with compartment syndrome received fasciotomies; 3 patients had objective compartment pressures.	4 postoperative infections occurred, including 1 secondaryto the fasciotomy procedure.
Cowin et al, 1998 [24]	Single-center, retrospective chart review using diagnosis codes for snakebites over a 3-year period. Some upper extremity bites were evaluated in a hand surgery clinic or by telephone for outcome data.	73 patients, 20 (74%) male. 27 (37%) lower extremity bites and 46 (63%) upper extremity. 24 pygmy rattlesnake bites, 11 diamondback rattlesnake, 15 cottonmouth, 9 coral snake.	No patient-level data reported; 3 patients diagnosed with compartment syndrome.	9/27 (33%) lower extremity and 22/46 (48%) upper extremity bites received antivenom. All 3 patients diagnosed with compartment syndrome received fasciotomy.	4/14 (29%) patients seen in clinic reported residual pain and tissue atrophy at the bite site. One patient who underwent fasciotomy had numerous deficits noted on physical exam. Patients contacted by telephone (n=10) reported subjective numbness (7/10), local tissue loss (2/10), and stiffness (2/10).
Tanen et al, 2001 [7]	Single-center, retrospective chart review of bite patients admitted to a medical toxicology service over a 6-year period.	236 patients, 191 (81%) male; 138 (56%) over the age of 13; 142 (60%) upper extremity bites, 39% lower extremity bites. It took an average of 1.7 hours between the time of the bite and arrival at a healthcare facility, and 5.3 hours on average from bite to antivenom infusion.	14% of children and 24% of adults developed hemorrhagic bullae. Compartment syndrome was diagnosed in 8 (3.3%) patients. Compartment pressures were only reported in one patient (80 mm Hg). Diagnosis was based on clinical signs included coldness to the touch and pulselessness in the other cases.	ACP administered to 77% of patients. An average of 28.5 vials were given. Fasciotomy performed on 3 patients, digital dermotomy on 5 patients	Mean hospital stay was 2.5 days, no long-term outcomes reported.
Tokish et al, 2001 [25]	Five-center, retrospective chart review of hospital admissions following snake bite over a 5-year period	163 patients, 89 (55%) male. Mean age 29 (range 1–81). 55% of bites were to the lower extremities, with one torso bite. 12% were intoxicated when bitten, and 29% were purposefully handling a snake. 98% of bites were from rattlesnakes. 10 (6%) of patients were treated with the “cut and suck” prehospital intervention, 7 (4%) used a constriction band, and 6 (4%) used a tourniquet.	6 (4%) patients developed compartment syndrome, and 16 (11%) developed necrosis in the inoculation site.	90% of patients received antivenom, with an average of 19 vials (range 0–75). The 6 patients with compartment syndrome received a fasciotomy, 1 patient received a finger amputation, and the 16 patients with necrosis all received surgical debridement. Surgery was more common in those receiving prehospital interventions such as incision and venom suction.	Mean hospital stay of 2.8 days.
Shaw et al, 2002 [26]	Single-center, retrospective chart review of pediatric bite patients over a 10-year period.	24 pediatric patients, 18 (75%) male. 14 (58%) upper extremity bites, 10 (42%) lower extremity bites.	2 patients developed necrosis of the tips of the digits. One patient developed compartment syndrome of the leg when antivenom administration was stopped after 5 vials due to an urticarial reaction. Anterior and posterior compartment pressures were 45 mm Hg.	Patients received an average of 12.5 vials of ACP antivenom except for one patient who received 4 vials of FabAV, then 5 vials of equine antivenom. The 2 patients with necrosis of the tips of the digits underwent limited debridement. One patient with compartment syndrome of the leg underwent fasciotomy.	Mean hospital stay of 3 days (range 1–10).
Campbell et al, 2007 [27]	Single-center, retrospective chart review of bite patients over a 10-year period	114 pediatric patients, 68% male. Mean age 4.2 years (range 1–17). 71 (62%) lower extremity bites. 65 (57%) copperhead, 9 (8%) rattlesnake, and 7 (6%) cottonmouth bites.	Compartment syndrome diagnosed in 2 (1.8%) patients. Compartment pressures in both patients exceeded 60 mm Hg. One patient was bitten by a cottonmouth, and the other by a copperhead.	7 (6%) patients received FabAV antivenom, 2 patients with compartment received fasciotomies.	No patient outcomes reported.
Correa et al, 2014 [28]	Single-center, retrospective chart review of pediatric patients envenomated over a 6-year period.	151 pediatric patients, 150 (66 %) male. 91 (60%) lower extremity bites, 58 (38%) upper extremity bites, 1 (1%) groin bite, 1 (1%) face bite. 65 copperhead, 5 cottonmouth, 3 coral snake, 3 pit viper, 1 pygmy rattlesnake, 1 fer de lance, and 1 timber rattlesnake bite.	Study used internal bite-severity scale, but patient-level data not reported. At least 2 (1.3%) patients diagnosed with compartment syndrome.	52 (34%) patients received antivenom (FabAV) with a median dose of 6 vials (range 1–16). 4 patients had surgery, and there was no significant difference between patients treated with antivenom and those not treated with antivenom. The operations included 2 fasciotomies for compartment syndrome, 1 full thickness skin graft, and 1 wound debridement. No mention of pressures.	Median hospital stay was 2 days.
Theilen et al, 2014 [29]	Single-center, retrospective chart review of surgical outcomes of patients after a snake bite in an academic referral center over a 4-year period	45 patients, no other demographic data reported.	No patient-level data reported.	36 (80%) received antivenom, with 16 (35.6%) requiring additional dosing. One case involved a minor dermotomy of the finger. 16/19 adult patients only required monitoring in the ED.	Mean hospital stay of less than 2 days.
Darracq et al, 2015 [30]	Retrospective case series from a statewide (California) poison center database over an 11-year period. Bites where fasciotomy was either discussed or performed were reviewed.	105 patients. 28 (27%) patients underwent fasciotomy, with 79% being male and 68% being upper extremity bites. Of the 74 cases where fasciotomy was discussed but not performed, 77% were male and 68% were upper extremity bites.	Compartment pressures were only recorded in 2 patients receiving fasciotomy and were elevated in both (36 and 70 mm Hg).	In patients receiving fasciotomy, a median of 4.5 vials of FabAV antivenom was preoperatively and 13.5 vials postoperatively, vs. a median of 18 vials in the group that did not receive a fasciotomy.	Length of stay was significantly longer in patients receiving fasciotomy (6.15 vs 3.45 days).

*ACP*, antivenin Crotalidae polyvalent; *FabAV*, Crotalidae polyvalent immune Fab; *mm Hg*, millimeters of mercury; *ED*, emergency department.

Case reports included a total of 12 patients with an age range of 1–59 years; 9/12 (75%) were male (Table 1). Species reported were the following: copperhead (3/12, 25%); western diamondback rattlesnake (2/12, 17%); great basin rattlesnake (2/12, 17%); cottonmouth (1/12, 8%); eastern diamondback rattlesnake (1/12, 8%); pygmy rattlesnake (1/12, 8%); and black-tailed rattlesnake (1/12, 8%). Bites were inflicted on the hands (4/12, 33%), dorsal foot (2/12, 17%) and anterior lower extremity (2/12, 17%). All females (3/3) suffered lower extremity bites.

Signs and symptoms reported included the following: rapid progression of swelling and edema (11/12, 92%); firm compartments (10/12, 83%); and pain (9/12, 75%). Erythema was not as commonly reported (3/1225%, Table 2). Compartment pressures were reported for all 12 patients, with a median compartment pressure of 60 millimeters of mercury (interquartile range [IQR] 55–68). All patients received antivenom. In six (50%) cases the authors did not specify which antivenom was used. For analytic purposes, we assumed that case reports from the 1980s and 1990s^11^^–^^13^ employed ACP and that another report from 2011^17^ used FabAV. In cases employing ACP, the median number of vials employed was 10 (IQR 7–15) and in cases employing FabAV the median was 21 (IQR 15–32). Fasciotomy was performed in 8/12 (66%) cases: 3/5 ACP and 4/6 FabAV (Table 3). With both antivenoms, patients undergoing fasciotomy received fewer vials than those who did not receive surgical management, keeping in mind the small number of patients in each group. Two patients who underwent fasciotomy reported motor deficits, compared to zero patients treated with medical therapy alone.

Chart review publications included data from 947 patients (Table 4). Three (33%) studies reported the snake species involved. Eight (89%) reported the location of bite. Only one (11%) study reported physical examination findings. Four (33%) studies reported specific compartment pressures. In total, 49 (5.2%) patients were diagnosed with compartment syndrome, and 44 of those patients underwent fasciotomy. Of patients who received fasciotomies, only six (12%) had objective compartment pressures reported. Although the chart reviews inconsistently reported which antivenom was used, it was assumed that publications from before 2001 employed ACP. The incidence of compartment syndrome in chart reviews from the ACP era was 8.3% (42 compartment syndrome diagnoses from 508 cases) compared to 1.6% (seven compartment syndrome diagnoses from 439 cases) after 2001. The number of vials of antivenom administered and information regarding the temporal association between antivenom administration and fasciotomy was not consistently reported.

## DISCUSSION

After an intensive screening process, we included 19 articles in this review. Most of the included retrospective cohort studies did not report individual patient-level data. Venom-induced compartment syndrome is a rarely reported disease process, as we identified only 88 cases consisting of 12 from case reports and 76 cases from larger retrospective reviews despite reviewing more than 40 years of literature. While the true prevalence is likely to be higher than the number of published reports, this nonetheless represents a small number in comparison to the approximately 6,000 snake envenomations occurring each year in the US.^29^

A clinically salient theme apparent in the data is that VICS portends better outcomes in comparison to trauma-induced compartment syndrome. In this review, only two patients from the included case reports (Table 3) were recorded to have residual motor deficits following VICS treatment, both of whom received a fasciotomy. No cases in the included published literature led to amputation or were associated with death. In contrast, following diagnosis and treatment of trauma or reperfusion-associated compartment syndrome, motor deficits range from 18–44%,^30^^,^^31^ and amputation rates range from 5.7–12.9%.^31^^–^^34^ While the pathology underlying venom-induced vs traumatic compartment syndrome is very different, the expected clinical course and functional outcome are important points to address when counseling patients at the bedside. It should be noted that follow-up times reported were variable and generally quite short—on the order of days to weeks; so patients’ final functional outcome(s) are unknown, identifying an important gap in the snakebite literature.

One interesting juxtaposition that became apparent during analysis was how the data differs between the articles published during the ACP and FabAV time periods. FabAV was approved for use in 2000, and the manufacture of ACP was discontinued in 2001. Looking at the case reports (Tables 1–3), four patients with compartment syndrome underwent fasciotomy in each antivenom “era”: ACP and FabAV. The median number of antivenom vials employed in the ACP (pre-2001) reports was 10 vials, while the median number of vials in patients receiving FabAV was 21. The manufacturer of ACP recommended an initial dose for severe envenomation of 10–15 vials with additional antivenom as needed based upon the clinical response.^35^ Considering real-world experience, a retrospective study of 414 patients treated for presumed rattlesnake envenomation reported a mean dose of 38 vials.^36^ The prescribing information for FabAV recommends an initial dose of 4–6 vials, followed by an additional 4–6 vials if needed to gain initial control of the envenomation, and an additional two vials every six hours for 18 hours (total dose of 14–18 vials).^37^ Clinical experience suggests that most patients achieve control of swelling with this regimen; additional dosing, when required, is typically directed toward hematotoxicity.^9^^,^^38^

The relatively low median ACP dose and somewhat high median FabAV dose noted in our review may reflect early discontinuation of ACP due to hypersensitivity reactions or fear of serum sickness, both of which are far less common with FabAV.^36^ This pattern aligns with a National Poison Data System review that revealed increased clinician use of antivenom, especially following *Agkistrodon* genus envenomation, after the year 2000.^29^ Alternately, this could indicate a premature decision to proceed with surgical management, prior to appropriate dosing of antivenom, during the ACP era. Expanding on this theme, analysis of the chart review studies (Table 4) revealed that the incidence of compartment syndrome in patients receiving ACP was 8.3% compared to 1.6% in patients receiving FabAV. While this decrease could reflect differences in the culprit snake species, or publication bias, as reports of VICS may no longer be considered novel or worthy of publication, the studies cited in Table 4 were generally comprehensive reviews of all snakebite patients evaluated by a center or physician group, not just the most interesting or severely envenomated patients. Therefore, it is plausible that the incidence of VICS may indeed be lower than prior to the introduction of FabAV, reflecting adequate control of tissue injury with appropriately dosed antivenom rather than fasciotomy.

Lastly, there was inconsistent reporting of data, particularly among the larger retrospective chart review studies. Laboratory abnormalities were also rarely reported but when they were, definitions of certain derangements such as coagulopathy and hypofibrinogenemia tended to differ between institutions precluding any analyses of or conclusions regarding these values. Although objective compartment pressure measurement prior to surgical management is the expert-recommended practice,^39^^,^^40^ many studies did not record these values. Based on the data available to us we could not determine whether these values were not obtained or simply not reported in articles.

## LIMITATIONS

As we conducted a scoping review, we did not perform a formal assessment of the included articles’ methodologies or risk of bias.^41^ We had access only to published articles and did not have access to original datasets. Without access to identifying information, it is possible that some of the case reports were also included in the retrospective chart-review studies. Although we employed a systematic search strategy, it is possible that we did not cast a wide enough net and that some studies meeting inclusion criteria were missed. Additionally, we only searched for published research that included cases of diagnosed compartment syndrome and did not analyze the clinical characteristics of patients who were not diagnosed with compartment syndrome. While objective measurement of compartment pressures is recommended, compartment syndrome is ultimately a clinical diagnosis that may vary between physicians, particularly those from different specialties (eg, medical and surgical), and it is possible that the diagnosis may be over- or under-reported depending on the author of each paper.

Many data points were scarcely reported, including laboratory values, compartment pressures, and vials of antivenom administered. It was also sometimes difficult to discern the order of events, particularly the timing of evaluations and interventions including antivenom administration, measurement of compartment pressures, and fasciotomy. Also, none of the included cases used the recently introduced Crotalidae immune F(ab')2 antivenom, and we are unable to comment on its possible efficacy in VICS. These limitations highlight the importance of rigorous, prospective data collection and reporting through centralized, enduring databases such as the North American Snakebite Registry.

## CONCLUSION

Compartment syndrome following North American snake envenomation is a rare disease process, and heterogeneity exists in its treatment despite global evidence discouraging fasciotomy. The seemingly increased tolerability of FabAV compared to ACP and the relatively positive short-term outcomes following suspected venom-induced compartment syndrome supports liberal antivenom usage, proceeding to fasciotomy only after careful clinical assessment with compartment pressure measurement and toxicology consult. Additionally, no amputations or deaths were reported in the reviewed articles. We illuminate several significant gaps in the literature, including the need for prospective studies assessing differences in long-term outcomes between treatment modalities, as well as the ideal timing of antivenom employment and subsequent fasciotomy.
